# Caraway as Important Medicinal Plants in Management of Diseases

**DOI:** 10.1007/s13659-018-0190-x

**Published:** 2018-10-29

**Authors:** Mohaddese Mahboubi

**Affiliations:** Medicinal Plants Research Department, Research and Development, TabibDaru Pharmaceutical Company, Kashan, Iran

**Keywords:** Caraway, *Carum carvi*, Obesity, Functional dyspepsia, Preservative, Carvone, Limonene

## Abstract

*Carum carvi* or caraway is traditionally used for treatment of indigestion, pneumonia, and as appetizer, galactagogue, and carminative. Essential oil, fixed oil and many other valuable extractive compounds with industrial applications are prepared from caraway. This review article has new deep research on caraway as medicinal plant. For preparing the manuscript, the information was extracted from accessible international databases (Google scholar, PubMed, Science direct, Springer, and Wiley), electronic resources and traditional books by key word of caraway or *Carum carvi*. The results of traditional studies exhibited that the galactagogue and carminative effects of caraway fruits are superior to other effects. Although, the traditional scholars used it as appetizer, while caraway was the main ingredient of anti-obesity drugs in traditional medicine, which has been confirmed in two modern clinical trials of human studies. Caraway oil in combination with peppermint oil or menthol is used for treatment of functional dyspepsia in clinical studies. Caraway oil topically on abdomen relieves the IBS symptoms in patient. Although, the use of caraway oil is not recommended in adults under 18 years due to insufficient data, but it can topically use as anti-colic and carminative agent in children or infants. The anti-aflatoxigenic, antioxidant and antimicrobial effects of caraway oil along with its reputation as spice help the industries to use it as natural preservatives and antioxidant agents.

## Introduction

*Carum* genus has 25 species, which *Carum carvi* or caraway is the only annual and biennial economical one as spice, aperitif, and carminative in food and pharmaceutical industries. Caraway is widely used in food products due to its pleasant flavor and preservative properties. Caraway fruits are used as remedy to cure indigestion, pneumonia, and as carminative, appetizer, and galactagogue in different traditional systems [1, 2]. According to European Union herbal monograph, caraway is traditionally used for symptomatic relief of digestive disorders (bloating and flatulence). Caraway fruits are used as popular remedy to mask alcoholic breath, anemia, and as antidote agent against venomous beats. Caraway fruits are used for flavoring of rye bread and its infusion is a remedy for colic and digestive disorders, and to fight worms [3]. Caraway fruits possess stimulant, expectorant and antispasmodic effects and is used for stomach aches, constipation, and nausea. It increases the secretion of gastric juice and promotes the discharge of bile, which increases the appetite and has digestive stimulatory effects [4]. Caraway is recommended by Persian traditional scholars to relieve the flatulence. It acts by improvement of digestive systems and deletion of accumulated gas from gastrointestinal tract, humors from stomach, which also relives the abdominal pain. For this purpose, the powder containing ajwain (*Trachyspermum ammi*), anise (*Pimpinella anisum*), cumin (*Cuminum cyminum*) and caraway with little rock candy is used daily as three teaspoons [5]. Ibn Sina traditionally used caraway for weight loss [6], stomach ache, burping, flatulence and intestinal spasms [7]. Caraway fruits are the main part of Safoof-e-Mohazzil, which is traditionally used as weight loss compound [8]. In Iranian folk medicine, caraway seeds are believed to possess the antiepileptic effects [9]. Caraway leaves with garlic are used for treatment of inflamed eczema by Indonesian people. Caraway tea is secreted in the milk with anti-griping effects on digestive tracts of breast feeding babies [10]. An ointment containing powdered fruits in Vaseline is used for animal’s scabies. Caraway fruits contain valuable therapeutic greenish-yellow essential oil (3–7%), which is used in many therapeutic formulations from ancient times. Caraway oil and thyme oil or caraway oil alone in ethanol (15 mL) and castor oil (150 mL) are used for scabies and mycosis [11, 12]. For supporting the digestive function and relief of colic and flatulence, a few drops of caraway oil are added to olive oil and topically are rubbed over the pit of stomach or abdomen [4, 13]. Although, two review articles have been published [7, 8] on pharmacological activities of caraway, but the difference of our review with others is the deepness of our views on caraway. For preparing the manuscript, the information was extracted from accessible international databases, electronic resources (PubMed, Science Direct, Springer, Wiley and Google), and traditional books (Persian or English modern traditional books), by key word of caraway or *Carum carvi*.

## Chemical Composition of Caraway

Caraway fruits contain essential oil (3–7%), fatty acids (10–18%) (petroselinic, linoleic and oleic acids), protein (20%), carbohydrate (15%) [14], phenolic acids (caffeic acids), flavonoids (quercetin, kaempferol) [15, 16]. Tannins, alkaloids and terpenoids were present in caraway aqueous extract [17]. Caraway oil is present in all parts of plant, but its concentration is highest in its fruits and is extracted by hydro-distillation method. Caraway chaff oil is produced by hydro-distillation of husks and stalks and contains less carvone and more terpenes. Caraway oleoresin is prepared from crushed dried caraway fruits by solvents (hexane ethanol, ethyl acetate, ethylene dichloride), which has greenish shade of yellow color, normally contain essential oil (20–25%), and fixed oil (60–75%). Environmental conditions have essential effects on seed quality, in other word, hot and dry weather is associated with significant low quality fruits [18]. Changes in quality of fruits mean the variation in main and major components of plant.

Fatty acids such as petroselinic, linoleic and oleic acids contain 10–18% of caraway fruits. A comparative study on fatty acid contents of caraway fruits among Tunisian, German and Egyptian ecotypes exhibited that the Tunisian chemotype had the higher total fatty acid composition (7.3%), followed by German (5.7%) and Egyptian (2.9%) ecotypes. Petroselinic acid was the major fatty acid in three ecotypes with percent of 31.1%, 30.9% and 29.5% in Tunisian, German and Egyptian caraway fruits. The corresponding linoleic acid contents were 28.7%, 30.0% and 29.2% in these oils. Oleic acid was present about 27.5%, 21.6%, 21.2% in Tunisian, German and Egyptian caraway fruit fixed oil. Myristic acid, palmitic, stearic and linoleic acids were present at low levels in fatty oils. The unsaturated fatty acids were higher in Tunisian ecotype (87.8%), followed by German (82.9%) and Egyptian (80.8%) samples. Saturated fatty acids (12.1–19.2%), monounsaturated (50.5–56.2%), polyunsaturated (30.2–31.6%) acids were the chemical profile of fatty acids [19]. Total fatty acid content of Tunisian annual caraway fruits from three ecotypes varied from 2.95 to 5.68% (w/w). Saturated (myristic, palmitic and stearic acids), monounsaturated (petroselinic acid), polyunsaturated (linoleic acid) fatty acids were present in three ecotypes [20]. l-fenchone (55.0%), p-methoxy benzaldehyde (19.2%) and p-methoxy allyl benzene (9.4%) were identified in caraway fixed oil (yield 4.5%), which is extracted by soxhlet apparatus with petroleum ether [21]. A significant reduction in fatty acid content, seed yield and growth parameters was observed under water deficient [22].

Carvone and limonene are two major components of oil, which account for 95% of essential oil compounds. According to European Pharmacopeia, caraway fruit should contain 3% essential oil with d-carvone (50–65%), and (+)-limonene (up to 45%) as main components and it contains less than 1.5% carveol and dihydrocarveol. d-carvone as the main component of caraway [23] is responsible for caraway odor reminiscent [24].

Many different factors have essential effects on yield and chemical compositions of caraway essential oil. Harsh conditions (heat and pH) have little effect on carvone and limonene content of essential oil, while the changes in minor components are very major [25]. Harvest time before maturation lead to lower essential oil content than that of full ripeness of caraway fruits. Also, the amount of carvone enhances during and after maturation [26]. The higher limonene to carvone ratio is proposed to be responsible for better quality of caraway. Increasing the storage time decreases the essential oil’s content and limonene to carvone ratio [27].

The limonene and carvone content increase under water deficit. The yield of fruit essential oil increases under water deficit [22]. Drying the seeds and reduction in fruit grinded powder size increase the essential oil yield [3]. Method of essential oil extraction, grinding methods, harvest time change the essential oil yields and their chemical composition. Extraction of oil from whole seed results in high content of carvone, while grinded fruits and their full ripeness result in high essential oil yield [28]. The use of ultrasound method for extraction of caraway oil results in higher content of carvone than limonene. Carvone and limonene yields were 1.3–2 times higher in ultrasound assisted extraction method, depending on temperature, than hydro-distillation method [29]. The reason is destructive mechanical effects of ultrasound method on fruits cell walls, which increases the leakage of cell content, while in hydro-distillation method, the plant extracts diffuses across glandular walls and causes cell rupture over a long time. Ultrasound treatment of caraway fruit result in release of essential oil after 30 min of extraction with the same yield for untreated fruit (1.72% vs. 1.68%) [30].

Limonene (48.1%), carvone (32.9%), and myrcene (7.9%) were the main components of caraway fruit essential oil, extracted by hydro-distillation assisted by microwave, while limonene (41.7%), carvone (55.8%), and myrcene were present in accelerated steam distillation assisted by microwave method [31]. Hydro-distillation by direct induction heating assisted by magnetic field (DIHMF) and by water and 6% food salt as electrolyte solutions resulted in 2.11% (w/w) essential oil, where limonene (7.4%) and carvone (85.2%) were its main components. The essential oil yield for hydro-distillation in presence of 6% food salt were 2.58%, with limonene (18.7%), and carvone (74.9%) as the main components [32]. Therefore, the isolation of essential oil by DIHMF is as efficient as hydro-distillation method, because of shorter time and higher quality of limonene and carvone. Carvone (57.7%) and limonene (35.5%) were two major components from ten identified compounds from Iranian caraway essential oil by using ultrasonic assisted with headspace solid phase micro-extraction (UA-HS-SPME) method [33]. The yields of essential oils obtained by hydro-distillation and microwave-assisted hydro distillation methods from Chinese caraway fruits were 4.7% and 4.2%, respectively. Limonene (43.5%), carvone (32.6%), and apiole (15.1%) were the main components of caraway fruits essential oil extracted by hydro distillation method, while limonene (48.4%), carvone (31.1%) and apiole (12.3%) were the main components of oil extracted by microwave-assisted hydro-distillation method [34].

The organ part of caraway causes changes in essential oil yield and its chemical profile. Dried ripped fruit caraway oil by hydro-distillation method consists of germacrene D (75%), caryophyllene, elemene, humulene, germacrene A and B, and two cadinenes. Germacrene B (51%) was the main component of seedlings root oil, which decreases during development [35].

Geographical location (Table 1) of gathered caraway fruits had greater effect than genotype on chemical composition of essential oil, while genotype had greater effects than location on morphological traits of fruits [36].

**Table 1:** Chemical composition of Caraway oil from different geographical region

Country	Yield (w/w)	Main components	References
German caraway essential oil	1.21%	Carvone (77.3%), and limonene (16.15%)	[19]
Tunisian chemotype	1.41%	Carvone (76.3%), and limonene (19.52%)	[19]
Egyptian chemotype	0.48%	Carvone (61.58%), limonene (29.11%), β-myrcene (3.97%) and α-selinene (10.9%)	[19]
Egyptian chemotype	–	Limonene (53.4%), β-selinene (11.1%), β-elemene (10.1%) and caryophyllene oxide (9.8%)	[40]
Chinese caraway	4.7%	Limonene (43.5%), carvone (32.6%), and apiole (15.1%)	[34]
Uttarakhand Himalaya, India	3.3–4.8%	Carvone (65.77–78.8%), and limonene (19.38–31.64%)	[37]
Commercial caraway	0.6–5.4%	Carvone (44.5–95.9%), limonene (1.5–51.3%), β-myrcene (0–0.4%), trans-dihydrocarvone (0–0.5%), and trans-carveole (0–0.2%)	[38]
Tunisian ecotype	0.86–1.20%	Carvone (76.78–80.53%), and limonene (13.05–20.29%)	[20]
Iran	–	γ-Terpinene (17.86%), cuminaldehyde (22.1%), γ-Terpinene-7-al (15.41%), p-cymene (7.99%)	[39]

A comparative study on essential oil yields and their chemical compositions of different ecotypes exhibited that the yields for essential oil extractions were 1.41%, 1.21% and 0.48% for Tunisian and German and Egyptian ecotypes. Carvone (61.6–77.4%) and limonene (16.2–29.1%) were the main components of caraway oils. Carvone (77.3%), and limonene (16.2%) were the main components of German caraway essential oil, while the corresponding values were 76.3% and 19.5% for Tunisian chemotype. Carvone (61.6%), limonene (29.1%), β-myrcene (3.9%) and α-selinene (10.9%) were the main components of Egyptian chemotype [19]. Limonene (43.5%), carvone (32.6%), and apiole (15.1%) were the main components of thirty-four components from Chinese caraway seeds essential oil extracted by hydro-distillation method [34]. The essential oil yield from caraway cultivated in five locality of Uttarakhand Himalaya, India were 3.3–4.8%. Carvone (65.77–78.8%), and limonene (19.38–31.64%) were the essential oil’s main components [37].

The essential oil yields varied from 0.6 to 5.4% for twenty commercial caraway samples from different countries. Carvone (44.5–95.9%), limonene (1.5–51.3%), β-myrcene (0–0.4%), trans-dihydrocarvone (0–0.5%), and trans-carveole (0–0.2%) were present in these essential oils [38].

**Fig. 1:**
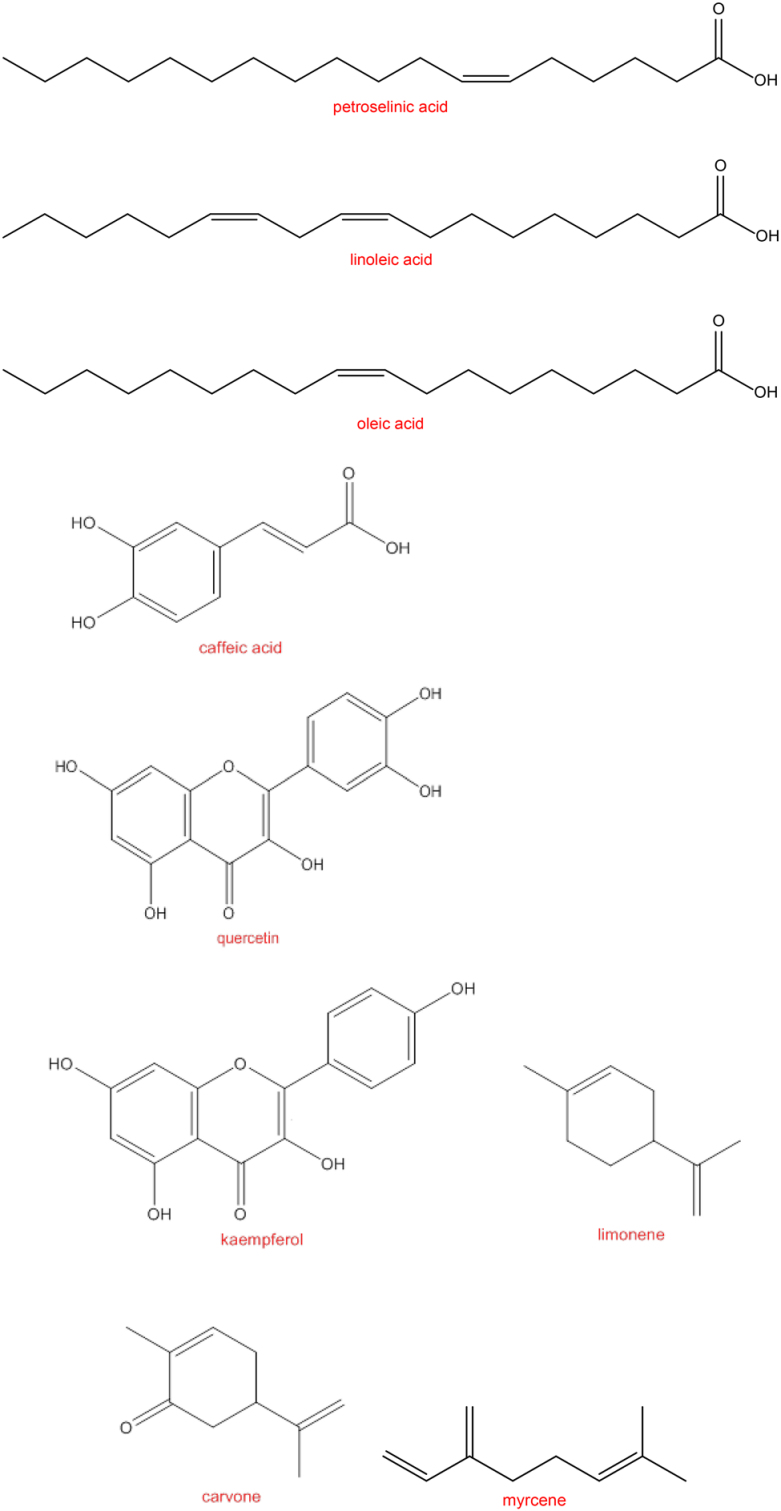
The chemical structure of main components of caraway fruits

Essential oil yields from three Tunisian ecotypes were in the ranges of 0.86%–1.20% (w/w). Carvone (76.8–80.5%) and limonene (13.1–20.3%) were the main components of 41 volatile compounds [20]. γ-Terpinene (17.86%), cuminaldehyde (22.1%), γ-Terpinene-7-al (15.41%), and p-cymene (7.99%) were the main components of caraway oil from Iran [39]. Therefore, due to the effects of many factors on chemical composition of essential oil and the amounts of each compounds, consideration to chemical composition of caraway oil is inevitable (Fig. 1).

## Biological Activities of Caraway Essential Oil

### Antimicrobial Effects of Caraway

The antimicrobial activity of caraway essential oil has been the subject of some in vitro studies. Egyptian caraway essential oil with limonene (53.4%), β-selinene (11.1%), β-elemene (10.1%) and caryophyllene oxide (9.8%) as the main components showed antibacterial activity against *Staphylococcus aureus*, *Salmonella typhi*, *Candida albicans*, *Aspergillus niger* and *Listeria innocua* with MIC value lower than 1 µg/mL. *Pseudomonas aeruginosa* had less sensitivity to Egyptian caraway essential oil (MIC > 16 µg/mL) [40]. Caraway oil with limonene (46.8%), and carvone (52.3%) as main components showed the MIC value of 18.8 ± 10.3 µL/mL against clinical isolates of *S. aureus* (n = 14) from skin lesions of patients. The MIC value of caraway essential oil on *S. aureus* ATCC 29213 was 2.1 ± 0.9 µL/mL [41]. Pullulan films (10%) containing caraway essential oil (0.12–10%) with carvone (52.2%), limonene (43.5%) inhibited the growth of *Salmonella enteritidis*, *S. aureus, Saccharomyces cerevisiae*, and *Aspergillus niger* on baby carrots samples. *S. enteritidis* was the most resistant microorganism among the others. Caraway essential oil inhibited *S. cerevisiae*, *Candida krusei* (MIC = 0.06, MFC = 0.12%), *S. aureus*, *B. subtilis*, *Penicillium expansum* (MIC = 0.12%, MLC = 0.12%), followed by *S. enteritidis*, *Escherichia coli* and *A. niger* with MIC and MLC values of 0.12% and 0.25% [42]. Caraway essential oil showed anti-Candidal effects against *C. albicans* isolates with MIC and MFC values of 0.03–0.11 and 0.06–0.11 mg/mL, respectively [43]. γ-Irradiation had no effect on major compounds of essential oil and its antibacterial activities. Cumin aldehyde, γ-terpinene, γ-terpinene-7-al and *p*-cymene had no change after irradiation. The antibacterial effects of treated and untreated caraway oil were the same against *B. subtilis*, *S. aureus*, *E. coli* and *P. aeruginosa* [44]. Caraway oil showed antibacterial activity against *S. aureus* (1 mg/g), and *E. coli* (10 mg/g) [45].

Caraway essential oil with γ-terpinene (17.9%), cuminaldehyde (22.1%), γ-terpinene-7-al (15.4%), and *p*-cymene (8.0%) significantly inhibited aflatoxin production without any effects on *Aspergillus parasiticus* growth (IC_50_ 621.9 and 56 µg/mL for AFB1 and AFG1, respectively) [39].

Topical caraway hydro-alcoholic extract with γ-terpinene (37.2%), cumin aldehyde (18.4%), *p*-cymene-7-ol (13.6%) and *p*-cymene (10.9%) showed the high antibacterial activity against *S. epidermidis* (MIC = 0.39, MBC = 1.56 mg/mL), *S. intermedius* (MIC = 0.76, MBC = 1.56 mg/mL), followed by *Streptococcus sanguinis* (MIC = 1.56, MBC = 3.125 mg/mL), *S. mitis* and *Campylobacter* spp. (MIC = 1.56, MBC = 6.25 mg/mL), respectively [46]. Phenolic compounds from defatted caraway powder exhibited antibacterial effects against *B. cereus*, *S. aureus*, followed by *E. coli*, and *S. typhimurium* [47]. The results of above studies imply on the antibacterial, antifungal and anti-candidal effects of caraway oil, although its main components play an important role in its antimicrobial effects. The antimicrobial effects of caraway essential oil has a negative correlation with carvone content, while limonene content of essential oil has positive correlation with antibacterial activities of essential oil [48]. Caraway is an inhibitor of P-gp efflux pumps [49]. It is recommended to evaluate the antiviral and anti-dermatophyte effects of caraway oil.

### Antioxidant Activity of Caraway

Free radicals are the reason of many ailment in the body or deterioration of compounds in different industries, so the antioxidant of compound is valuable [50].

Γ-irradiated caraway essential oil significantly reduced DPPH radicals higher than trolox (20.7% vs. 12.6%). The inhibitory effects of γ-irradiated caraway essential oil on peroxidation process of linoleic acid were not affected from irradiation [44].

The protective effects of oral caraway oil (10 mg/kg) on gentamicin induced nephrotoxicity has been confirmed in rat animal model. Caraway essential oil reduced the increased plasma malondialdehyde (MDA) in rats exposed to gentamicin. It also increased superoxide dismutase (SOD), catalase (CAT) and glutathione peroxidase (GSH-Px) in rats [51]. The enhancing effects of oral caraway oil (5, 10, 20 mg/kg) on serum level of GSH-Px, CAT and SOD and the decreasing effects on serum level of MDA in streptozotocin induced diabetic rats were confirmed. In other word, caraway oil has reducing effect on the oxidative stress in diabetes mellitus [52]. The preventive effects of 100 mg/kg essential oil against cecal ligation and puncture model, which induced septic related oxidative liver injury in rats showed myeloperoxidase (MPO) activity and TBARS were suppressed by essential oils in cecal ligation and puncture model of rats. Caraway oils significantly reverse the depleted hepatic cellular Glutathione (GSH) comparable with 10 mg/kg indomethacin. Caraway essential oils had significant suppressive effects on aspartate transaminase (AST) and alanine transaminase (ALT). Plasma alkaline phosphatase (ALP), total bilirubin, albumin and total protein were not affected from caraway essential oil. Essential oils reduced infiltration and sequestration of PMN, interstitial edema, congestion, necrosis and the severity of liver injury index in sepsis model of rats, comparable to indomethacin [53]. The reno-protective effects of oral caraway essential oil (10 mg/kg) in streptozotocin induced diabetic rats were shown by increasing in GSH-Px to normal levels of control group and significant improvement in pathology of diabetic nephropathy with minor pathological changes in glomerular lobulation, mild cellular infiltration in interstitial tissue [54]. Caraway (30, 60 and 90 mg/kg) for 30 weeks reduced the levels of intestinal, colonic and caecal tissue lipid peroxidation products (conjugated dienes, lipid hyperoxides, TBRAS) and increased the antioxidant enzymes such as SOD, CAT, GSH, glutathione reductase of rats with colon carcinogenesis. Caraway seed extract reduced the activity of tissue GSH-Px, glutathione S transferase (GST), ascorbic and α-tocopherol levels. Caraway fruits could decrease the tumor size and tumor incidence. Caraway make resistant the tumor cells to free radical attack, which result in reduction of cell proliferation of cancerous cells. The activation of antioxidant enzymes scavenge the free radicals in colon cancer rats [55]. The protective effects of 150 mg/kg oral caraway seed aqueous extract on liver, kidney and fertility exposed to cadmium toxicity in rat animal model was confirmed. Caraway Seed extract decreased the AST and ALT enzymes levels. The serum level of creatinine had the same value with control group. A significant increase in total antioxidant capacity and a significant reduction in serum levels of MDA was observed in caraway female rats. The histopathological samples of liver from caraway treated rats showed normal structures with strong fuchsinophilic reaction in hepatocytes. Pretreatment of cadmium treated rats with caraway seed extract improved the histopathological structure of kidney tubules. An improvement in liver functions was observed after treatment of cadmium exposed albino rats with caraway seed extract, which maintain the liver enzymes in normal level. Caraway seed extract had ameliorative effects against hepatic histological changes induced by cadmium [56].

Topical application of caraway hydro-alcoholic extract on oral mucositis induced by 5-fluorouracil in golden hamsters improved histological parameters in 5-fluorouracil induced mucositis, which is associated with reduction in oxidative stress of oral mucosa [46]. Cold-pressed caraway fixed oil with high total phenolic content showed high radical scavenging activities in different antioxidant system with inhibitory effects on human LDL oxidation [57]. Caraway with high total phenolic content had been good reducing power in different in vitro antioxidant system [58]. It has been confirmed high level of flavonoids [59] and steroid like substances of caraway inhibited CYP1A1 at mRNA level, which generates reactive metabolites that are toxic or carcinogenic metabolites with binding property of attachment to DNA [60].

### Anti-diabetic Effects of Caraway

Oral caraway essential oil (10 mg/kg) decreased the fasting blood sugar in streptozotocin induced diabetic rats [54]. Streptozotocin induced diabetic rats treated with oral doses of caraway oil (5, 10, 20 mg/kg) had higher weight and lower level of blood glucose in comparison with control group [52]. The effects of caraway oil on serum blood glucose [52] and no effectiveness of plasma level of insulin from caraway oil imply that reduction in serum level of glucose is regulated by a mechanism independent of insulin secretion [61]. The antioxidant effects of caraway oil may involve in anti-diabetic effects of caraway oil.

### Anti-inflammatory Effect of Caraway

Oral caraway essential oil (10 mg/kg) reduced the pro-inflammatory cytokines (IFN-γ, IL-6 and TNF-α) in rats exposed to gentamicin [51]. Oral and intra-peritoneal administrations of caraway hydro-alcoholic extract (100, 200, 400 mg/kg) and its essential oil (100, 200, 400 μL/kg) in an immunological model of colitis of rats induced by trinitrobenzene sulfonic acid (TNBS) reduced colon tissue lesions and colitis index, comparable to prednisolone and Asacol^®^. Caraway extracts and its essential oil reduced the inflammation and infiltration of white blood cells in mucus and sub-mucosal layers [62]. The immunological effects of caraway has been confirmed [63].

Therefore, caraway can be a good candidate for management of inflammatory diseases such as Irritable Bowel Syndrome (IBS), and Inflammatory bowel disease (IBD).

### Anticonvulsant Effects of Caraway

The antiepileptic effects of caraway [9] have been the subject of study according to folk believes. Caraway aqueous extract (200, 400, 800, 1600 and 3200 mg/kg i.p) and essential oil (25, 50, 100, 200 and 400 mg/kg, i.p) in comparison with diazepam (3 mg/kg) in pentylenetetrazole (PTZ) induced convulsion of mice animal model dose dependently increased the latency time the onset of myoclonic and clonic seizures. Caraway oil or its extract had preventive effect against tonic seizure and PTZ induced death. The anticonvulsant effects of essential oil was higher than its aqueous extract. Essential oil or extract had no effect on neuromuscular coordination. Therefore, relaxation of muscle was not involved in its anticonvulsant effects [17].

### Diuretic Effects of Caraway

The ripe fruits of caraway is used as diuretic in Moroccan Traditional Medicine. The diuretic effects of acute and sub-chronic oral dose of 100 mg/kg caraway seed aqueous extract was compared with 10 mg/g furosemide in normal male Wistar rats. Caraway seed extract increased the urine output, total volume of excreted urine, urinary level of K^+^ and Na^+^ comparable with furosemide in except of urinary K^+^ level. The plasma K^+^ and Na^+^ was not affected from caraway seed extract. The administration of caraway seed aqueous extract for 8 days showed strong diuretic effects and virtually had no effect on urinary K^+^ excretion for the entire 8 days. Caraway seed extract had no renal toxicity during 8 days of treatment [64].

### Anti-glycemic Effects of Caraway

Caraway seed aqueous extract decreased the cholesterol and triglycerides in normal and streptozotocin diabetic rats. Six hours after oral administration of 20 mg/kg caraway seed aqueous extract, the plasma level of triglycerides and cholesterols of normal rats decreased. Oral single dose of caraway seed extract decreased the plasma cholesterol levels only in streptozotocin induced rats. No change was observed in plasma triglycerides concentration in caraway treated streptozotocin induced rats. Repeated oral administration of caraway extract for 15 days significantly reduced the plasma triglycerides in normal and streptozotocin rats [65].

### The Effects of Caraway on Reproductive Organs

The modulation effects of caraway on sexual function and fertility in females have been claimed in Egyptian folk medicine. 150 mg/kg oral caraway seed aqueous extract on fertility organ exposed to cadmium toxicity in rat animal model increased the serum estrogen in proestrus phase of cadmium treated rats. Atretic follicles and minor degenerative changes, stratified uterine epithelium and cystic endometrial gland with weakly reacted glands was observed in caraway treated rats, which exposed to cadmium. Caraway fruit aqueous extract showed antifertility effects, it significantly increased the estrogen level and reduced the progestrone and FSH levels at proestrus phase [56]. Caraway aqueous or ethanol extracts from doses higher than 200 mg/kg showed estrogenic effects. After 30 days administration of caraway extracts, changes in vaginal smear, was observed. The ovary weight, uterus weight and estrogen levels increased, while gonadotropins levels decreased [66].

### Other Biological Activities of Caraway

The pharmacokinetic properties of paracetamol (oral, intraperitoneal) are affected from chronic intake of caraway oil [67]. Administration of 100 mg caraway with rifampin, isoniazid and pyrazinamide in 20 healthy volunteers increased the plasma levels of antibiotics. Caraway increased the bioavailability indices C_max_ of antibiotics and area under curve (AUC) [68]. Caraway enhanced the action of rifampicin [69], isoniazid and pyrazinamide in rats [69, 70]. KCl (80 mM) related tonic contraction and the phasic contraction to acetylcholine (320 nM) of rat isolated uterus dose dependently was inhibited by caraway essential oil, which implicates to its usefulness for control of uterus spasm [71]. Feeding the lactating cow with 0.2 and 1 g caraway oil/kg dry matter for 24 days had no effect on food consumption, methane emission and milk production. The milk had higher fresher aroma and lower stored aroma [72]. Caraway oil had no effect on growth promotion and feed intake in weaning piglet [73]. Caraway oil especially carvone is a good potato sprouting inhibitor [74]. The molluscicidal activity of caraway seed powder, ethanol extract with LC_50_ 140.58 mg/L after 96 h and 130.61 mg/L after 24 h was confirmed against snail *Lymnaea acuminate* and may be due to limonene content [75].

## The Efficacy of Caraway in Clinical Trials of Human Diseases

### Caraway in Management of Thyroid Hormones

Caraway increased the TSH level in patients with papillary thyroid carcinoma under treatment with fixed dose of levothyroxine. 40 mg/kg/day caraway capsule and 100 µg/day levothyroxine in a hypothyroidism patients increased TSH level, 2 weeks after ingestion of caraway. Five months after discontinuation of caraway, the TSH level returns to normal level. T_4_ and T_3_ levels decreased by consumption of caraway [76]. The enhancing effects of caraway on T_3_ and T_4_, and decreasing effect on TSH [77] and its anti-hypothyroidism effects stimulate metabolic rate, which reduces body fat and body weight [78].

### Caraway in Management of Obesity

According to ancient Unani medicine texts, obesity (Saman-e-Mufrat) means excessive fat, which accumulates in the body. It is a phlegmatic disease, which balgham predominates in the body. Phlegm with blood produces viscosity of the blood and constrict blood vessels. Deposition of fat (Atherosclerosis) obstructs passage of oxygen in the organs, which can cause death. It is recommended to obese patients to eat 5 g Safoof-e-Muhazzil with 20 mL Arq-e-Zeera (caraway water or distillate of caraway in order to gathering the water containing soluble essential oil), twice a day. There are some clinical studies, which evaluates its efficacy.

In randomized, triple blind, placebo-controlled clinical trial, the efficacy of caraway water on weigh loss of women with BMI 25–39.9 kg/m^2^ was evaluated in comparison with placebo. Caraway water was prepared by hydro-distillation of 1 kg caraway fruits and producing 10 L caraway water. Placebo was containing 1% g/L caraway essential oil. The patients in each group are recommended to use 30 mL intervention or placebo, 20 min before lunch for 3 months. The patients were visited every week from the beginning until the end of treatment. The regimen program and any probable side effects were recorded during the study. Clinical assessments on vital parameters along with blood and urine tests and biochemical parameters were evaluated. The mean weight, BMI, waist circumference, waist-to-hip ratio and body fat percent significantly reduced in caraway water compared to placebo group. The body muscle percent increased in caraway water group. Vital clinical symptoms including heart rate, systolic and diastolic blood pressure, urine specific gravity and lipid profile had no significant difference with placebo group [79]. A significant reduction in appetite level and carbohydrate intake was observed after 90 days treatment with caraway extract. Caraway extract had no significant effect on fat and protein intake of the overweight subject, compared to control group. All of the anthropometric indices (waist circumference, waist to hip ratio, thigh circumference, and mid-upper arm circumference) exhibited a significant reduction in caraway oil, compared with placebo group. The average appetite level significantly reduced in caraway extract group [80]. The limitation of two studies are placebo group. Indeed caraway water is a kind of diluted caraway oil, which is used as placebo in this study.

### Caraway in Management of Functional Dyspepsia

The most important application of caraway is its use as remedy for digestive problems. In one clinical trial, the efficacy and tolerability of enteric-coated capsule containing caraway oil and menthol was compared to placebo in Functional Dyspepsia patients (Rome III criteria) taking their usual medications (PPIs, H2RAs, anticonvulsants, beta blockers, antihistamines, antidepressants/TCAs, pain modulators, and antiacids). Each capsule was containing 25 mg of caraway oil and 20.75 mg l-menthol. The patients took two capsules of intervention or placebo, twice daily, 30–60 min before a meal in the morning and at dinner time for 28 days. Changes in Global Overall Symptom and Clinical Global Impressions, safety and tolerability were examined. The results of study showed that the reduction of symptoms was numerically superior in intervention group than that of placebo. 61% and 49% of patients found intervention and placebo treatments “good” or “very good” in improvement of Clinical Global Impressions (p = 0.23). No serious adverse events were reported in intervention group [81].

In a prospective, double-blind, multicenter trial, 114 outpatients with chronic or recurrent functional dyspepsia, the efficacy of peppermint-caraway-oil preparation (n = 58) or placebo (n = 56) was evaluated on improvement of abdominal pain and discomfort. The patients took capsules, twice daily, in the morning and dinner. 8.6% and 5.4% in intervention and placebo groups left the study, before the end of treatments. A significant improvement in symptoms was observed in intervention group compared to placebo group (p = 0.0004). The average symptom scores reductions were 7.6 ± 4.8 (62.3%) and 3.4 ± 4.3 (26%) in intervention and placebo groups (p < 0.0001). The corresponding at least 10% improvements were 86.2 and 57.1%, respectively. Disease-specific symptoms, quality of life (QoL) in patients with functional dyspepsia symptoms improved consistent with epigastric pain syndrome and postprandial distress syndrome [82].

The efficacy of caraway oil (50 mg) and peppermint oil (41.5 mg) versus placebo over a treatment period of 28 days in reduction of Global Overall Symptoms (GOS) and Clinical Global Impressions (CGI) of patients suffering from functional dyspepsia were evaluated. At the end of treatment, PDS (78%) and EPS (72%) patients in intervention group reported the treatment “good” or “very good” in CGI assessment, while the corresponding values in placebo group were 50% (PDS) (p = 0.09) and 40% (EPS) (p = 0.046), respectively. The sensations of pressure, heaviness, fullness, reduction in epigastric pain or discomfort symptoms improved no significantly compared to placebo group [83].

The effect of capsule containing 25 mg of caraway oil and 20.75 mg of l-menthol versus placebo for functional dyspepsia symptoms was evaluated after 24 h. In this study, 100 subjects suffering from functional dyspepsia (Rome III criteria) took two capsules of intervention or placebo in the morning and at dinner time. The patients were categorized into PDS or EPS predominant based on their symptoms and took their routine treatments. The self-reported Global Overall Symptoms (GOS) were used in patients at 24 h. At 24 h, a significant (p= 0.04) reduction in PDS symptoms and significant improvement in EPS symptoms (p =  0.076) in the overall population were observed [83].

Enteric-coated and non-enteric-coated capsules, which contain 90 mg peppermint and 50 mg caraway oil had effects on the migrating motor complex of healthy volunteers and decreased the number of contractions and contraction amplitudes during the various phases of the migrating motor complex. The effects of non-enteric-coated capsule was mainly during the first migrating motor complex after administration, while the effects of enteric-coated capsule temporally had delayed during the second migrating motor complex. Both capsules were safe and had local smooth muscle relaxing effect [84].

Evaluating the efficacy of enteric-coated capsule containing 90 mg peppermint and 50 mg caraway oil (twice daily) on 118 patients suffering from functional dyspepsia in comparison 30 mg cisapride daily on the base of pain score (VAS), frequency of pain and dyspeptic symptom score (DSS) showed that intensity of pain decreased from 6.6 ± 1.3 to 2.0 ± 2.2 in enteric-coated capsule group. The corresponding values were 6.5 ± 1.3 and 1.9 ± 2.3 at baseline and after twenty-ninth day in cisapride group. The mean reduction in frequency of pain and DDS was comparable in two groups after 29 day (1.9 ± 2.2 vs. 2.0 ± 2.5 and 12.7 ± 14.0 vs. 13.2 ± 14.3, respectively). Twelve and 14 patients in enteric-coated capsule and cisapride experienced non-serious adverse effects, which diarrhea was reported as the most frequent symptoms [85].

In a prospective, randomized, placebo controlled multicenter trial, the effect of enteric-coated capsule on dyspeptic symptoms and life quality of male with functional dyspepsia after 2 and 4 weeks of evaluation on the base of pain score, discomfort score, Nepean Dyspepsia index symptom score, and Nepean Dyspepsia index total scores showed that enteric-coated capsule was superior to placebo in reducing all variables. Pain score, the discomfort score and Nepean Dyspepsia index symptom score (p < 0.000l) and Nepean Dyspepsia index total score (p = 0.0037) improved significantly compared to placebo. The treatment was well tolerated and improved the quality of life in patients [86]. For management of functional dyspepsia, caraway oil is used in combination with menthol or peppermint oil.

### Caraway in Management of Irritable Bowel Syndrome (IBS)

In randomized controlled cross-over trial, the efficacy of hot poultice made with caraway poultice in treatment of IBS for 3 weeks, followed by 2 week ‘wash-out’ phases, was compared with hot or cold poultices of olive oil. Forty-eight patients with diarrhea dominant IBS were in this study. Two-third of patients applying caraway oil poultice found the treatment good or very good. A significant difference in symptom severity were found among caraway oil poultice and cold poultice. The response rate was 43.9%, 20% and 18.9% for caraway oil, hot or cold olive oils, respectively. The IBS-QOL total score, subscales health worry and dysphoria, Bristol stool scale had significant difference in caraway oil with the others. The treatment with caraway oil was without any adverse effects. The adequate relief was reported 51.8%, 23.5% and 25.8% for caraway oil, hot or cold olive poultices [87]. Caraway is the active components of Iberogast as remedy for treatment of gastro-intestinal motility, and as anti-inflammatory, anti-oxidant agents, which inhibit the gastric acid production [88].

## Daily Dose of Caraway

0.15–0.3 mL of essential oil in three divided daily dose is recommended for adults and elders. The oral administration of caraway essential oil is not recommended for children and adolescents under 18 years old, during lactation and pregnancy. Semisolid preparations of caraway oil in concentration of 2% can be applied daily as a thin layer on the abdominal area of infants, children, adolescents, adults and elders [89]. Caraway fruits (1.5–6 g) or its essential oil (0.15–0.3 mL) are described as carminative, spasmodic gastro-intestinal complaints, bloating, flatulence and sensation of fullness. 0.3 mL of caraway oil is corresponded to 273 mg of caraway oil (density 0.91 g/mL). Caraway aqueous or ethanol extracts from doses higher than 200 mg/kg showed estrogenic effects [66].

## Contraindication and Precaution

The use of preparation containing caraway oil on broken skin, around the eyes or mucous membranes and in the patients with liver disease, achlorhydria, cholangitis, gallstones or other biliary disorders is not recommended [89]. Caraway fruit or caraway oil is not recommended to use during pregnancy and lactation due to insufficient data. A warning of use in patients with obstruction of bile ducts, liver diseases, cholangitis, gallstones or other biliary diseases is present due to complete inhibitory effects of caraway on gallbladder emptying in healthy humans [90]. The contraindication with inflammation of kidneys was reported and overdoses of caraway oil for long time causes kidney and liver damage [4].

## Toxicity of Caraway

The acute oral and dermal LD_50_ of caraway oil in rats and rabbits were 3.5 and 1.78 mL/kg, respectively. Pure caraway oil had no irritating effects on the backs of hairless mice. Applying 4% caraway oil in petrolatum on 25 human subjects had been no irritating and sensitization reactions effects in a 48 h closed patch test [91].

In the ESCOP monograph, the acute oral LD_50_ of caraway oil in rats was reported from two different studies as 3.5 and 7.4 mL/kg, respectively. The acute dermal LD_50_ of caraway oil in rabbits was reported as 1.8 mL/kg. Intra-peritoneal, intravenous LD_50_ of d-carvone in mice were 482.2 and 1500 mg/kg, the oral LD_50_ of d-carvone in rats and guinea pigs were 1640 and 766 mg/kg. ADI (Acceptable Daily Intake) for d-carvone was 0–1 mg/kg/day [90].

In a randomized, triple-blind, placebo-controlled study, the safety of caraway aqueous extract was evaluated on 35 overweight and obese healthy women compared with placebo. The patients received 30 mL of caraway water or placebo (diluted caraway essential oil) for 12 weeks, and the general health status, urine test, blood pressure, heart rate and blood chemistry were evaluated. No adverse events were reported after 12 weeks of treatment. Heart rate, liver, kidney functions were not affected from interventions. A significant difference in red blood cell and platelet distribution width levels were observed among two groups. A significant increase in red blood cell and significant reduction in platelet distribution width were observed in caraway water, which imply that the possible beneficial effect of caraway seed aqueous extract for the treatment of anemia. Hyperthyroidism is associated with an reduction of platelet distribution width [78]. Caraway is well tolerated in therapeutic doses and showed no toxic effects toward human [10]. Acute toxicity of caraway exhibited the maximum nonlethal dose of caraway essential oil and aqueous extract were 400 and 3200 mg/kg, respectively [17]. The established ADI for d-carvone is 0.6 mg/kg bw/day [92].

## Concluding Remarks and Future Directions of Research

The review article has a deep view on caraway fruits as famous medicinal plants in different pharmaceutical, food and cosmetic industries. Caraway fruits are used in different traditional systems as curative plants for management of different ailments, especially for management of digestive disorders. In traditional medicine, the galactagogue and carminative effects of caraway fruits are superior to other biological effects. Although, traditional scholars use caraway fruits as appetizer in foods, but caraway fruits or its water are one important ingredient of anti-obesity drugs in Unani traditional medicine [93]. There are two parallel clinical trials, which confirmed the anti-obesity effects [78, 80] of caraway water (Zeereh-Aragh) in comparison with placebo. Caraway reduces the plasma triglycerides and cholesterol levels in normal and streptozotocin rats [65] and also it enhances T_3_ and T_4_ levels. All above mechanisms along with its decreasing effect on TSH [77], are proposed the activation of metabolism in the body. The important role of caraway oil in management of functional dyspepsia has been confirmed in many clinical studies, but caraway oil always is used in combination with peppermint oil or menthol in enteric coated pills [85, 86]. The topical use of caraway oil around the abdomen has relieved the IBS symptoms in patient [87]. Although, the use of caraway oil is not recommended for adults under 18 days due to insufficient data, but it can be topically used as anti-colic and carminative agent for children or infantile. Although, caraway fruits are used for treatment of many human disorders according to traditional believes, the antiepileptic, anti-inflammatory, galactagogue effects of caraway oil have been confirmed in pre-clinical studies. Due to the high yield of essential oil, the use of caraway oil as antioxidant [50, 59] and preservatives [94] in food industries are recommended. Caraway could act as bioenhancers [49, 95]. Evaluating the safety of topical creams as abdominal pain reliever in children should be considered. The anti-aflatoxigenic, antioxidant and antimicrobial effects of caraway oil along with its reputation as spice help the industries to use it as natural preservatives and antioxidant agents instead of synthetic ones.

## References

[1] Rasooli I, Allameh A, Preedy VR (2016). Chapter 32—caraway (*Carum carvi* L.) essential oils. Essential oils in food preservation, flavor and safety.

[2] Malhotra S (2006). *Caraway*. Handbook of Herbs and Spices.

[3] Attokaran M (2017). Natural food flavors and colorants.

[4] Peter K (2006). Handbook of herbs and spices.

[5] Larijani B, Esfahani MM, Moghimi M, Shams Ardakani MR, Keshavarz M, Kordafshari G, Nazem E, Hasani Ranjbar S, Mohammadi Kenari H, Zargaran A (2016). Iran. Red Crescent Med. J..

[6] Nasser M, Tibi A, Savage-Smith E (2009). J. R. Soc. Med..

[7] Johri R (2011). Pharmacogn. Rev..

[8] Agrahari P, Singh DK (2014). J. Biol. Earth Sci..

[9] Gorji A, Khaleghi Ghadiri M (2001). Neurosci. Biobehav. Rev..

[10] Németh É (2003). Caraway: the genus Carum.

[11] Pruthi JS (2001). Minor spices and condiments: crop management and post-harvest technology.

[12] Duke JA (2002). Handbook of medicinal herbs.

[13] A. Ożarowski, W. Jaroniewski, J. Muszyński, *Rośliny lecznicze i ich praktyczne zastosowanie*, edn. (Instytut Wydawniczy Związków Zawodowych, 1987)

[14] Olennikov DN, Kashchenko NI (2014). Khimiya Rastitel’nogo Syr’ya.

[15] Escop, E.S.C.o. Phytotherapy (2003). *ESCOP Monographs: the scientific foundation for herbal medicinal products*.

[16] Sachan AK, Das DR, Kumar M (2016). J. Chem. Pharm. Res..

[17] Showraki A, Emamghoreishi M, Oftadegan S (2016). Iran. J. Med. Sci..

[18] Aćimović M, Filipović V, Stanković J, Cvetković M, Đukanović L (2015). Ratarstvo i povrtarstvo.

[19] Laribi B, Kouki K, Bettaieb T, Mougou A, Marzouk B (2013). Ind. Crops Prod..

[20] Laribi B, Kouki K, Mougou A, Marzouk B (2010). J. Sci. Food Agric..

[21] Abdalaziz MN, Ali MM, Gahallah MD, Garbi MI, Kabbashi AS (2017). Int. J. Comput. Theor. Chem..

[22] Laribi B, Bettaieb I, Kouki K, Sahli A, Mougou A, Marzouk B (2009). Ind. Crops Prod..

[23] Ravid U, Putievsky E, Katzir I, Weinstein V, Ikan R (1992). Flavour Fragr. J..

[24] de Carvalho CCCR, da Fonseca MMR (2006). Food Chem..

[25] Yin Y, Zarghami N, Heinz D (1970). J. Food Sci..

[26] Sedláková J, Kocourková B, Lojková L, Kuban V (2003). Plant Soil Environ..

[27] Sedlakova J, Kocourkova B, Kuban V (2001). Czech J. Food Sci..

[28] Sedláková J, Kocourková B, Lojková L, Kubáň V (2003). Hort. Sci..

[29] Smain C, Ahcène L, Hamid A, Farid C (2004). Flavour Frag. J..

[30] Assami K, Pingret D, Chemat S, Meklati BY, Chemat F (2012). Chem. Eng. Process..

[31] Benkaci-Ali F, Mékaoui R, Scholl G, Eppe G (2014). World Acad. Sci. Eng. Technol..

[32] Rivera LL, Vilarem G (2007). Flavour Fragr. J..

[33] Meshkatalsadat MH, Salahvarzi S, Aminiradpoor R, Abdollahi A (2012). Dig. J. Nanomat. Biostruct..

[34] Jiang Z-T, Sun M-L, Li R, Wang Y (2011). J. Essent. Oil Bearing Plants.

[35] Wichtmann EM, Stahl-Biskup E (1987). Flavour Fragr. J..

[36] Solberg SO, Göransson M, Petersen MA, Yndgaard F, Jeppson S (2016). Biochem. Syst. Ecol..

[37] Gwari G, Bhandari U, Andola HC, Lohani H, Chauhan N (2012). Indian J. Nat. Prod. Res..

[38] Raal A, Arak E, Orav A (2012). J. Essent. Oil Res..

[39] Razzaghi-Abyaneh M, Shams-Ghahfarokhi M, Rezaee M-B, Jaimand K, Alinezhad S, Saberi R, Yoshinari T (2009). Food Control.

[40] Tarek N, Hassan HM, AbdelGhani SMM, Radwan IA, Hammouda O, El-Gendy AO (2014). Beni-Suef Univ. J. Basic Appl. Sci..

[41] Kwiatkowski P, Mnichowska-polanowska M, Pruss A, DziĘcioŁ M, Masiuk H (2017). Herba Pol.

[42] Gniewosz M, Krasniewska K, Woreta M, Kosakowska O (2013). J. Food Sci..

[43] Skrobonja JM, Delić DN, Karaman MA, Matavulj MN, Bogavac MA (2013). Zbornik Matice srpske za prirodne nauke.

[44] Fatemi F, Allameh A, Khalafi H, Rajaee R, Davoodian N, Rezaei MB (2011). J. Food Biochem..

[45] Kwiatkowski P, Giedrys-Kalemba S, Mizielińska M, Bartkowiak A (2015). Herba Polonica.

[46] Mardani M, Afra SM, Tanideh N, Tadbir AA, Modarresi F, Koohi-Hosseinabadi O, Iraji A, Sepehrimanesh M (2016). Oral Dis..

[47] Thippeswamy NB, Naidu KA, Achur RN (2013). J. Pharm. Res..

[48] Seidler-Ao ykowska K, dzia BK, KarpiDska E, Bocianowski J (2013). Acta Sci. Agron..

[49] Meher B (2016). World J. Pharm. Pharm. Sci..

[50] Suhaj M (2006). J. Food Compos. Anal..

[51] Erjaee H, Azma F, Nazifi S (2015). Vet. Sci. Dev..

[52] Erjaee H, Rajaian H, Nazifi S, Chahardahcherik M (2015). Comp. Clin. Pathol..

[53] Fatemi F, Allameh A, Khalafi H, Ashrafihelan J (2010). Appl. Radiat. Isot..

[54] N.H.A. El-Soud, N.A. El-Lithy, G. El-Saeed, M.S. Wahby, M.Y. Khalil, F. Morsy, N. Shaffie, (2014)

[55] Kamaleeswari M, Nalini N (2006). J. Pharm. Pharmacol..

[56] Abdel-Wahab A, Hashem Abdel-Razik AR, Abdel Aziz RL (2017). Asian Pac. J. Trop. Med..

[57] Yu LL, Zhou KK, Parry J (2005). Food Chem..

[58] Fatemeh B, Mahdi K, Javad K (2006). Int. J. Food Sci. Technol..

[59] Skrovankova S, Misurcova L, Machu L (2012). Adv. Food Nutr. Res..

[60] Naderi-Kalali B, Allameh A, Rasaee MJ, Bach HJ, Behechti A, Doods K, Kettrup A, Schramm KW (2005). Toxicol. In Vitro.

[61] Ene A, Nwankwo E, Samdi L (2008). J. Pharmacol. Toxicol..

[62] Keshavarz A, Minaiyan M, Ghannadi A, Mahzouni P (2013). Res. Pharm. Sci..

[63] Al-Snafi AE (2016). Immunol. Endocr. Metab. Agents Med. Chem..

[64] Lahlou S, Tahraoui A, Israili Z, Lyoussi B (2007). J. Ethnopharmacol..

[65] Lemhadri A, Hajji L, Michel JB, Eddouks M (2006). J. Ethnopharmacol..

[66] Thakur S, Bawara B, Dubey A, Nandini D, Chauhan NS, Saraf D (2009). Int. J. Phytomed..

[67] Samojlik I, Ðaković-Švajcer K, Božin B, Mikov M (2012). BMC Pharmacol. Toxicol..

[68] Choudhary N, Khajuria V, Gillani ZH, Tandon VR, Arora E (2014). Perspect. Clin. Res..

[69] Sachin BS, Sharma SC, Sethi S, Tasduq SA, Tikoo MK, Tikoo AK, Satti NK, Gupta BD, Suri KA, Johri RK, Qazi GN (2007). Phytother. Res..

[70] Sachin B, Monica P, Sharma S, Satti N, Tikoo M, Tikoo A, Suri K, Gupta B, Johri R (2009). Hum. Exp. Toxicol..

[71] Sadraei H, Ghannadi A, Takei-bavani M (2003). Int. J. Aromather..

[72] Lejonklev J, Kidmose U, Jensen S, Petersen MA, Helwing ALF, Mortensen G, Weisbjerg MR, Larsen MK (2016). J. Dairy Sci..

[73] Schone F, Vetter A, Hartung H, Bergmann H, Biertumpfel A, Richter G, Muller S, Breitschuh G (2006). J. Anim. Physiol. Anim. Nutr..

[74] Hartmans KJ, Diepenhorst P, Bakker W, Gorris LGM (1995). Ind. Crops Prod..

[75] Kumar P, Singh DK (2006). Chemosphere.

[76] Naghibi SM, Ramezani M, Ayati N, Zakavi SR (2015). Daru.

[77] Dehghani F, Panjehshahin M, Vojdani Z (2010). Iran. J. Veter. Res..

[78] Kazemipoor M, Radzi CWJBW, Hajifaraji M, Cordell GA (2014). Phytother. Res..

[79] Kazemipoor M, Hajifaraji M, Haerian BS, Mosaddegh MH, Cordell GA (2013). Evid. Complement. Altern. Med..

[80] Kazemipoor M, Hamzah S, Hajifaraji M, Radzi CWJBW, Cordell GA (2016). Phytother. Res..

[81] Chey WD, Lacy BE, Cash BD, Epstein M, Shah SM (2017). Gastroenterology.

[82] Rich G, Shah A, Koloski N, Funk P, Stracke B, Köhler S, Holtmann G (2017). Neurogastroenterol. Motil..

[83] Chey WD, Lacy BE, Cash BD, Epstein M, Shah SM (2017). Gastroenterology.

[84] Micklefield G, Greving I, May B (2000). Phytother. Res..

[85] Lin X, Orr WC, Chen J (2000). Gastroenterology.

[86] Holtmann G, Gschossmann JM, Buenger L, Wieland V, Heydenreich C-J (2001). Gastroenterology.

[87] Lauche R, Janzen A, Ludtke R, Cramer H, Dobos G, Langhorst J (2015). Digestion.

[88] Wegener T, Wagner H (2006). Phytomedicine.

[89] HMPC (2015) monograph on *Carum carvi* L., aetheroleum. EMA/HMPC/715094/2013. 30 Churchill Place, Canary Wharf, London E14 5EU, United Kingdom

[90] HMPC (2015) Assessment report on *Carum carvi* L., fructus and *Carum carvi* L., aetheroleum. EMA/HMPC/715093/2013

[91] Opdyke D (1973). Food Cosm. Toxicol..

[92] Committee ES (2014). EFSA J..

[93] Meena A, Brijendra S, Yadav A, Uttam S, Ramanjeet K, Ayushy S, Vertika G, Bhavana P (2010). J. Pharm. Res..

[94] Sun M, Jiang Z, Li R (2009). China Condiment.

[95] Tatiraju DV, Bagade VB, Karambelkar PJ, Jadhav VM, Kadam V (2013). J. Pharmacogn. Phytochem..

